# Air travel and incidence of pneumothorax in lymphangioleiomyomatosis

**DOI:** 10.1186/s13023-018-0964-6

**Published:** 2018-12-13

**Authors:** Cynthia Gonano, Jérôme Pasquier, Cécile Daccord, Simon R. Johnson, Sergio Harari, Violette Leclerc, Lucy Falconer, Eleonora Miano, Jean-François Cordier, Vincent Cottin, Romain Lazor

**Affiliations:** 1 Service de médecine interne, Hôpital neuchâtelois, La Chaux-de-Fonds, Switzerland; 2 Institut de médecine sociale et préventive, Centre hospitalier universitaire vaudois, Lausanne, Switzerland; 3 Service de pneumologie, Centre hospitalier universitaire vaudois, PMU BU44.07, Rue du Bugnon 44, 1011 Lausanne, Switzerland; 4 National Centre for Lymphangioleiomyomatosis, University of Nottingham, Nottingham, United Kingdom; 5 U.O. di Pneumologia e Terapia Semi-Intensiva Respiratoria, Servizio di Fisiopatologia Respiratoria ed Emodinamica Polmonare, Ospedale San Giuseppe, MultiMedica IRCCS, Milan, Italy; 6 Association France Lymphangioléiomyomatose (FLAM), Plouhinec, France; 7 LAM Action, Nottingham, United Kingdom; 8 Associazione Italiana Linfangioleiomiomatosi (A.I.LAM-ONLUS), Arco, Italy; 9 National Reference center for rare pulmonary diseases, Claude Bernard University Lyon 1, OrphaLung, UMR 754, Lyon, France

**Keywords:** Lymphangioleiomyomatosis, Incidence, Pneumothorax, Pleurodesis, Air travel

## Abstract

**Background:**

Pulmonary lymphangioleiomyomatosis (LAM) is a rare disease of women characterized by multiple lung cysts leading to respiratory insufficiency and frequent pneumothorax (PT). Air travel (AT) could increase the risk of PT in LAM through rupture of subpleural cysts induced by atmospheric pressure changes in aircraft cabin. To determine whether AT increases the risk of PT in LAM, we performed a retrospective survey of members of European LAM patient associations. A flight-related PT was defined as occurring ≤30 days after AT.

**Results:**

145 women reported 207 PT. In 128 patients with available data, the annual incidence of PT was 8% since the first symptoms of LAM and 5% since LAM diagnosis, compared to 0.006% in the general female population. Following surgical or chemical pleurodesis, the probability of remaining free of PT recurrence was respectively 82, 68, and 59% after 1, 5 and 10 years, as compared to only 55, 46 and 39% without pleurodesis (*p* = 0.026). 70 patients with available data performed 178 AT. 6 flight-related PT occurred in 5 patients. PT incidence since first symptoms of LAM was significantly higher ≤30 days after AT as compared to non-flight periods (22 versus 6%, risk ratio 3.58, confidence interval 1.40–7.45).

**Conclusions:**

The incidence of PT in LAM is about 1000 times higher than in the general female population, and is further increased threefold after AT. Chemical or surgical pleurodesis partly reduces the risk of PT recurrence in LAM.

## Background

Pulmonary lymphangioleiomyomatosis (LAM) is a rare disease affecting almost exclusively women in their reproductive age. It is characterised by the proliferation of abnormal smooth muscle-like cells (LAM cells) in the lungs and lymphatic system, and is considered as a low-grade metastasizing neoplasm [1–5]. In the lungs, LAM cell proliferation leads to the development of multiple thin-walled cysts and progressive destruction of the parenchyma resulting in dyspnea, obstructive ventilatory defect, reduced carbon monoxide transfer factor, and hypoxemia [6–10]. Another typical feature of pulmonary LAM is the occurrence of pneumothorax (PT), which affects more than half of patients during disease course with frequent relapses [6–9].

Air travel (AT) is a matter of concern in patients with LAM [11]. During commercial flights, the cruising altitude varies between 4′534 and 14′630 m, and aircraft cabin is pressurized to approximately 565 mmHg corresponding to an altitude of 2438 m. The resulting decrease in alveolar oxygen partial pressure may worsen pre-existing hypoxemia in patients with respiratory diseases. In addition, following Boyle’s law, when barometric pressure decreases during ascent, the air eventually trapped in a non-communicating space such as a pulmonary cyst may increase its initial volume by 30%, and could lead to overinflation and rupture, with consecutive PT [12–14]. The occurrence of a PT during flight may have serious consequences in patients with impaired lung function, and its treatment in such circumstances may be delayed. In 2 retrospective studies addressing the issue of AT in patients with LAM, the reported frequency of PT after AT was respectively 2.2 and 1.1% per flight [11, 15]. However, these studies did not determine whether AT by itself constitutes a risk factor for the occurrence of PT.

To explore this issue, we performed a survey of European LAM patients. The main objectives were to calculate the annual incidence rate of PT in LAM, and to determine whether AT increases the risk of PT in this population.

## Methods

### Patient recruitment

Patients with pulmonary LAM were recruited through European LAM patient associations in France, Germany, Italy, Spain, United Kingdom, and a rare lung disease registry in Switzerland. Isolated patients from other countries were also recruited on an individual basis. Data were collected retrospectively through a questionnaire available in the local language. Patients provided informed consent.

### Data collection

Patients were asked to provide detailed information regarding the date of first symptoms attributable to LAM, date of LAM diagnosis, current forced expiratory volume in one second (FEV1) if known, and the occurrence and date of lung transplantation. They were also asked to report details on each episode of PT, which occurred since the first symptoms of LAM, including date of PT, affected side, and treatment received according to pre-specified categories (spontaneous resolution, needle aspiration, chest tube, or chemical or surgical pleurodesis). Patients were also asked to report with the best possible accuracy the first 4 AT which took place since the first symptoms of LAM, including date, origin and destination, occurrence of PT during or after AT, and whether it occurred on a lung previously treated for PT. Patients were encouraged to provide dates with the best possible accuracy from personal archives (diaries, travel invoices), and obtain details on PT dates and treatments from their physician. The French patient association France Lymphangioléiomyomatose (FLAM) performed data collection and capture. An anonymized database was provided to the investigators. Patients who were identified by the survey as experiencing a PT ≤ 30 days after AT received a second questionnaire to ascertain that PT was diagnosed by a physician and by chest X-ray, the dates of AT and PT, and the treatments received.

### Data analysis

In a first set of analyses, we determined the overall incidence of PT in the study population. The beginning of exposure to the risk of PT was defined as the date of the first symptoms attributable to LAM, and in a second analysis as the date of LAM diagnosis. The end of exposure to the risk of PT was defined as the date of survey completion. When a PT was the first symptom attributable to LAM, it was included in the calculation of PT incidence during the exposure period, which started with the first symptom. We considered 2 different hypotheses to compute PT incidence: 1) the risk of PT is constant across the whole LAM population, 2) the risk of PT is variable from one patient to another. A standard Poisson regression (model 1) was used to compute the incidence according to the first hypothesis. To compute PT incidence according to the second hypothesis, we used 2 different regression models to estimate the variable risk [16]: a negative binomial regression (model 2), and a Poisson regression with a random intercept (model 3). In each of these models, only an intercept was considered (mean model). Model 1 is equivalent to calculate the ratio of the total number of observed PT and the sum of all exposure periods. In model 2, we hypothesized that the incidence was distributed as a gamma distribution (the negative binomial distribution can be viewed as a Poisson distribution where the parameter is itself a random variable distributed as a gamma distribution). In this model, the estimation of the intercept leads to an estimation of the mean incidence rate (over the patients). In model 3, we supposed that the intercept was normally distributed and therefore the incidence followed a log-normal distribution. In this model, the estimation of the intercept leads to an estimation of the median incidence rate. Patients were withdrawn from the analysis if the date of the first symptoms, the date of LAM diagnosis, or any date of PT were missing. Transplanted lungs were not considered at higher risk for PT and were withdraw from the calculation.

In a second set of analyses, we determined whether pleurodesis reduced the risk of PT in the study population. For this purpose, we compared the recurrence rate of PT after conservative treatment (spontaneous resolution, needle aspiration or chest tube) and after medical or surgical pleurodesis in patients who experienced a first episode of PT, using the Kaplan-Meier method. Each lung was considered as an independent observation. Patients were withdrawn from the analysis if any date of PT or the affected side were missing, or if they never experienced a PT.

In a third set of analyses, we determined whether AT increased the risk of PT occurrence in LAM. A PT was arbitrarily defined as related to AT if it was diagnosed by chest X-ray within 30 days after AT. This time interval was defined before the survey. Although cyst rupture related to barometric pressure change is expected to occur during AT, the resulting PT (i.e. the leak of a significant amount of air from the airspaces to the pleural cavity through the ruptured cyst) may be delayed, as suggested for another cystic lung disease, the Birth-Hogg-Dubé syndrome (BHD) [17]. An interval of up to 30 days has also been observed in a study on the occurrence of PT due to AT in BHD [18]. We also considered that a patient with mild respiratory symptoms may have seeked medical attention only after several days.

Each lung was considered as an independent observation. Patients were withdrawn from the analyses if any date of PT, the affected side, or any date of AT were missing. Based on our (see below) and previous findings [19] that pleurodesis was only moderately effective in reducing the risk of PT recurrence, lungs treated with pleurodesis were still considered at risk of PT.

An AT was defined as both an outbound and a return trip, each of which may have consisted of one or more stops, i.e. one or more episodes of ascent and descent. The time interval between outbound and return trips was not recorded, but we reasoned that in the vast majority of AT, the duration of a trip would be < 15 days. Indeed, according to European statistics, the average duration of trips performed by European citizen is 5.4 days [20], and 94% of trips last < 14 days [21]. Thus, the interval of 30 days after the outbound trip was considered appropriate to observe the occurrence of PT related to an AT. The date of AT, defined as the date of the first outbound flight, was considered as day 0.

To determine whether AT increased the risk of PT occurrence, we compared the incidence of PT during the 30 days following AT (days 0 to + 29) to the incidence of PT at all other periods, i.e. before AT (from first symptoms or diagnosis) and > 30 days after AT. A standard Poisson regression was used for these analyses. Quantitative data were expressed as mean and standard deviation (SD). Statistical analyses were performed with the R software version 3.4.4 [22].

## Results

### Study population

145 filled questionnaires were available. All patients were women. Their countries of origin were France (31.7%), Germany (23.4%), United Kingdom (17.2%), Italy (15.2%), Spain (5.5%), Switzerland (4.1%), Austria (0.7%), Belgium (0.7%), Ireland (0.7%), and Turkey (0.7%). The response rate, available for the French association, was 51%. The mean (SD) age at time of survey was 47 (12) years. The mean (SD) age was 36 (11) years at first symptoms attributable to LAM (*n* = 139), and 41 (11) years at LAM diagnosis (*n* = 145). The mean (SD) FEV_1_ at time of survey was 58 (24) % predicted (*n* = 71).

### Incidence of pneumothorax in LAM

Among the 145 patients, 6 had missing dates of first symptoms of LAM, and 11 had one or more dates of PT missing. In the remaining 128 patients, the mean follow-up duration since the first symptoms attributable to LAM was 11.4 years, and the cumulated follow-up duration was 1454 patient-years. The mean follow-up duration since LAM diagnosis was 6.4 years, with a cumulated follow-up duration of 817 patient-years.

The 145 patients reported a total of 207 PT. Eighty-three patients (57%) had at least one PT, and 56 (39%) had 2 or more PT. Among the 137 patients for whom the side of each PT was determined, the mean (SD) number of PT per lung was 1.8 (1.0).

In the 128 patients with available data, the annual incidence rate of PT since the first symptoms of LAM and since LAM diagnosis according to the 3 statistical models are shown in Table 1. Since the number of PT varied widely among patients, we considered that model 3 was the most appropriate to describe the incidence of PT. With this model, the annual incidence of PT was 8% since first symptoms of LAM, and 5% since LAM diagnosis. Higher rates were found with the 2 other statistical models (Table 1), showing that model 3 was the most conservative.

**Table 1:** Annual incidence rate of pneumothorax in LAM (*n* = 128)

Start of exposure period		Model 1	Model 2	Model 3
First symptoms of LAM		0.12 (0.1, 0.14)	0.18 (0.13, 0.24)	0.08 (0.05, 0.11)
LAM diagnosis		0.09 (0.07, 0.11)	0.11 (0.07, 0.15)	0.05 (0.03, 0.08)

Data were calculated from 3 models: Model 1: constant risk of PT across the whole LAM population, standard Poisson regression. Model 2: variable risk from one patient to another, negative binomial regression. Model 3: variable risk from one patient to another, Poisson regression with random intercept. Results are expressed as incidence per patient per year (95% confidence intervals)

### Efficacy of pleurodesis to prevent recurrence of PT

**Fig. 1:**
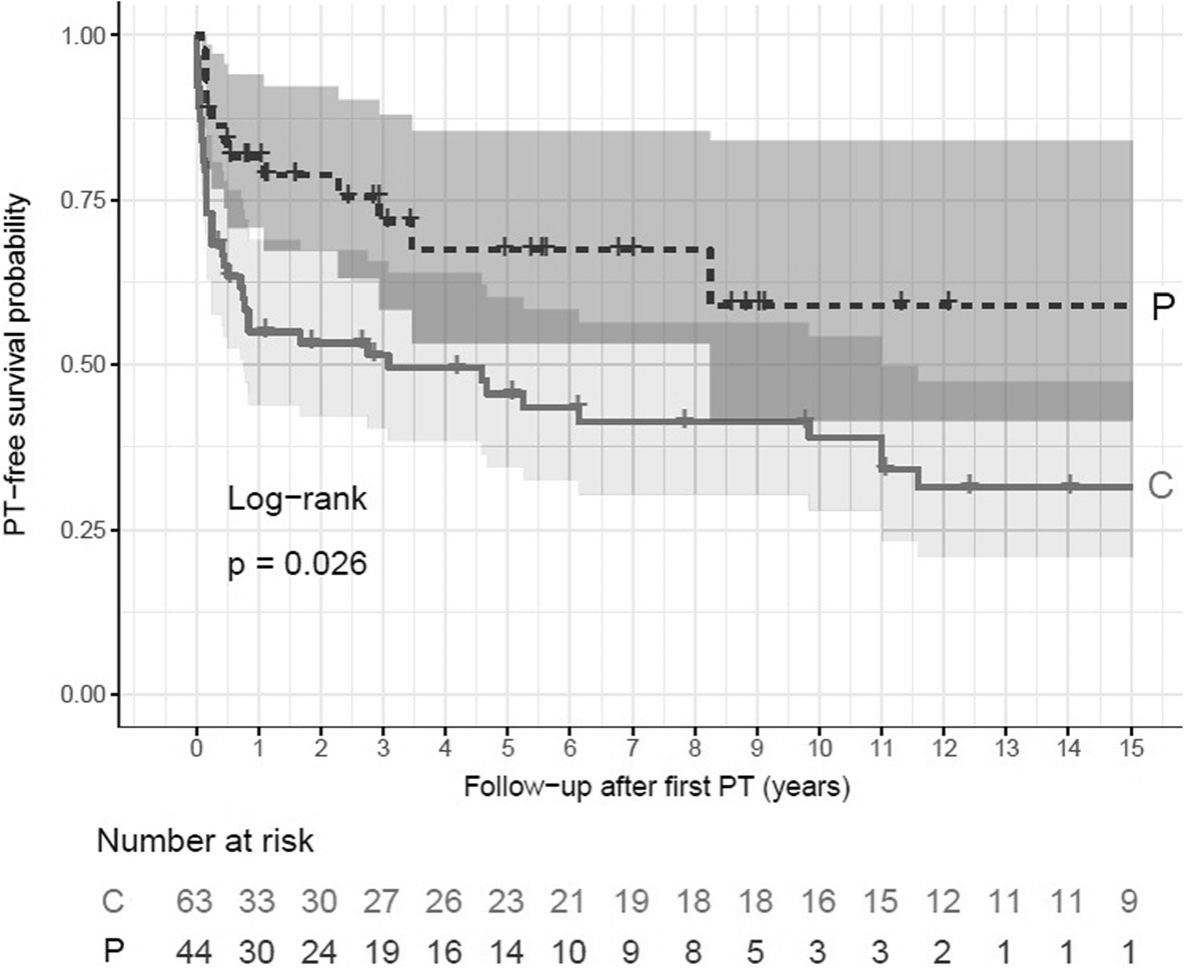
Probability of pneumothorax-free course after the first pneumothorax, according to treatment received for this pneumothorax. Each lung was considered as a separate observation. Probability of pneumothorax-free course was significantly lower after conservative treatment (C, grey solid line) than after chemical or surgical pleurodesis (P, black broken line) (*p* = 0.026, log-rank test). Grey areas reflect 5–95% confidence intervals. Data were censored at the date of questionnaire filling if a second pneumothorax did not occur. PT: pneumothorax

The probability of remaining free of PT recurrence without pleurodesis was 55% at one year, 46% at 5 years and 39% at 10 years after the initial PT, versus 82, 68 and 59% with pleurodesis, respectively (*p* = 0.026, Log rank, Kaplan-Meier method) (Fig. 1). No significant difference was observed between chemical and surgical pleurodesis (*p* = 0.69, data not shown). As the protective effect of pleurodesis was only partial, and for the purpose of calculating the risk of PT after AT, we considered that a lung treated with pleurodesis remained at risk of PT afterwards.

### Risk of pneumothorax after air travel

Eighty-three out of 145 patients (57%) travelled by air. Among them, 3 (cases 26, 36, and 79) had missing or incomplete dates of AT, 3 (cases 38, 53, and 89) had missing or incomplete dates of PT, and 4 (cases 11, 38, 89, and 93) had missing side of PT. In one other patient (case 141), a PT was reported on the same day as an AT, but this event was not counted since all AT (*n* = 3) and PT (*n* = 4) in this patient were reported as occurring on January 1st, which was considered very unlikely. Moreover, 4 patients had missing dates of first symptoms of LAM or did not fly after first symptoms of LAM (cases 31, 75, 97, 144). Thus, after removing these 13 cases, a population of 70 patients was used to determine the risk of PT associated with AT after the first symptoms of LAM. These 70 patients reported a total of 178 AT. Considering each lung as an independent observation, 140 lungs performed a total of 356 AT. After LAM diagnosis, 57 patients performed 139 AT, or 114 lungs performed 278 AT.

Six PT occurred in 5 patients (bilateral PT in one patient revealing the disease) within 30 days after AT (Table 2), including 4 patients who experienced their first PT at this occasion, and one who had 3 PT previously and relapsed on the same side despite previous chemical pleurodesis. Three other patients (cases 27, 98, and 107) mentioned the occurrence of PT after AT in comments, but since this was not consistent with the reported dates of PT and AT, these narratively mentioned PT were not counted. Overall, the rate of PT within 30 days after AT was 2.8% (5/178) per patient and 1.7% (6/356) per lung after first symptoms of LAM. After LAM diagnosis, the rate of PT within 30 days after AT was 2.2% (3/139) per patient and 1.1% (3/278) per lung.

**Table 2:** Characteristics of LAM patients who presented PT within 30 days after AT

Case number	Age at PT (years)	Number of previous PT	Side of current PT	Delay between AT and PT (days)	Perceived relationship between AT and PT	PT treatment
143	26	0	R	0	yes	surgical pleurodesis
143	26	0	L	0	yes	surgical pleurodesis
84	64	0	R	19	yes	chemical pleurodesis
25	26	0	L	24	yes	surgical pleurodesis
102	44	3	R	29	no	spontaneous healing
142	59	0	R	29	yes	surgical pleurodesis

*PT* Pneumothorax, *AT* air travel, *L* Left, *R* Right

The incidence of PT within 30 days after AT was compared to the incidence of PT during all non-flight periods since first symptoms of LAM, and since LAM diagnosis, respectively (Table 3). When using the first symptoms of LAM as the beginning of the period at risk, a significantly higher incidence of PT was found within 30 days after AT (0.223, CI 0.089–0.453) as compared to non-flight periods (0.062, CI 0.052–0.075) with a risk ratio of 3.58 (CI 1.40–7.45). When using the date of LAM diagnosis as the beginning of the period at risk, the incidence of PT was also increased within 30 days after AT (0.143, CI 0.035–0.370) as compared to non-flight periods (0.044, CI 0.032–0.059), but the difference did not reach statistical significance (risk ratio 3.25, CI 0.79–8.93). No PT was counted twice because of overlapping periods at risk.

**Table 3:** Incidence of PT during post-flight and non-flight periods

Start of exposure period	Exposure period type	Exposure period duration (lung-yr)	Number of PT during exposure period	PT incidence per lung per yr (CI)	Risk ratio (CI)
first symptoms of LAM	total	1835.4	119	0.065 (0.054–0.077)	
	non-flight	1808.6	113	0.062 (0.052–0.075)	3.58 (1.40–7.45)
	30 days post-flight	26.8	6	0.223 (0.089–0.453)	
LAM diagnosis	total	930.7	43	0.046 (0.034–0.061)	
	non-flight	909.7	40	0.044 (0.032–0.059)	3.25 (0.79–8.93)
	30 days post-flight	21.0	3	0.143 (0.035–0.370)	

*CI* confidence interval

## Discussion

In the present study, we determined for the first time the incidence of PT in LAM, which is about 1000 times higher than in the general women population. Another new finding is that the incidence of PT is increased threefold after AT compared to baseline incidence, suggesting that AT could be a risk factor for the occurrence of PT in LAM. Additionally, we confirm previous data showing that pleurodesis is partly effective in reducing the recurrence rate of PT in LAM [19].

Several series have reported that 50 to 80% of LAM patients experience PT during disease course [6–9], but the incidence of PT in LAM has not been determined previously. Based on the wide interindividual variation in the number of PT observed in the present study (range 0–4), we considered that the risk of PT would be variable from one patient to another, and as the median is more robust to outliers than the mean, model 3 was considered the most appropriate. With this model, the incidence of PT in the LAM population was 8% per year from the first symptoms of LAM, and 5% per year from LAM diagnosis. In comparison, the incidence of spontaneous PT in the general female population is 1 to 6/100′000 per year, or 0.001 to 0.006% [23]. Using the most conservative estimate, the incidence of PT in LAM is therefore about 1000 times higher than in the general population.

Only one retrospective survey has previously examined the efficacy of pleurodesis to prevent PT recurrence in LAM [19]. Among 301 episodes of first PT in 193 patients, the recurrence rate was 66% after conservative therapy, 27% after chemical pleurodesis and 32% after surgical pleurodesis [19]. In the present study, we confirm that pleurodesis significantly reduces the risk of PT recurrence in LAM (Fig. 1), and that chemical and surgical pleurodesis are of similar efficacy. However, the risk of recurrence after pleurodesis remained much higher than in spontaneous primary PT, with reported recurrence rates of 0 to 3.2% after surgical pleurodesis, and 2.5 to 10% after thoracoscopic talc poudrage [24]. Based on these findings, we considered that LAM patients with pleurodesis remain at risk of PT after AT, and did not exclude post-pleurodesis periods from calculations. Indeed, among the 5 patients who had a PT within 30 days after AT, one had a previous pleurodesis on the same side.

The incidence of PT during AT aboard commercial aircrafts in the general population is unknown but probably very low. Only 0.003% of passengers have an in-flight medical problem requiring emergency intervention [25], and PT is usually not mentioned in studies on in-flight emergencies [25–27]. However, there are several case reports of in-flight PT [14, 25, 28–30]. Furthermore, a relationship between atmospheric pressure changes and occurrence of spontaneous PT has been demonstrated in several studies [31–34]. A case of spontaneous PT triggered by an ascent of 350 m in a high-speed lift has also been reported [35]. In the US Air Force personnel, the incidence of spontaneous PT was 47/100′000/year [36], i.e. fourfold higher than in the US Navy [36] or the general male population [23]. Taken together, these data suggest that variations of atmospheric pressure during AT may trigger PT. Although the risk appears very low in the general population, it may be higher in subjects prone to PT, such as LAM patients. Lung function testing has also been shown to trigger PT in LAM patients with an incidence rate of 0.02 to 0.04/100 tests [37], which is certainly much higher than in the general population. To our knowledge, despite the extremely wide use of this procedure, only 3 cases of PT after lung function testing have been reported in the literature [38–40].

Two previous studies have analysed the occurrence of PT related to AT in LAM patients [11, 15]. The first study surveyed members of the LAM Foundation (USA) and the LAM Action registry (UK) who travelled by air. The rate of PT was estimated to 4% per patient and 2.2% per flight [11]. In another study of LAM patients who travelled to the National Institutes of Health, the rate of PT related to AT was 2.9% per patient and 1.1% per flight, compared to 1.3% per patient and 0.5% per journey with ground travel [15]. The rate of PT in the present study (2.8% per patient per flight and 1.7% per lung per flight) is consistent with these previous findings [11, 15], thus reinforcing the validity of our observations. However, these previous studies did not provide any information on a possible causal relationship between AT and PT because no comparison was made between patients who flew and those who did not fly [11, 15].

In contrast, and for the first time, we compared the incidence of PT after AT with the baseline incidence of PT in LAM, to assess the risk of PT attributable to AT. We observed a significant threefold increase of PT incidence ≤30 days after AT as compared to non-flight periods (22 versus 6% per year, risk ratio 3.58, CI 1.40–7.45) when using the date of first symptoms as the beginning of the period at risk. A similar threefold increase was observed when the period at risk was calculated from the date of diagnosis (14 versus 4% per year, risk ratio 3.25, CI 0.79–8.93), although the difference did not reach statistical significance due to a smaller sample size. We however believe that the date of first symptoms is more relevant than the date of diagnosis to define the period a risk in LAM, since PT is the first disease manifestation in about half of cases, and diagnosis is often delayed by several years [6, 7]. Indeed, in the present study, the mean age at first symptoms was 36 years, whereas the mean age at diagnosis was 41 years. Discarding the period between first symptoms and diagnosis would have led to miss a time span during which patients are already exposed to the risk of PT, as well as a significant proportion of events. Indeed, among the 5 patients who experienced PT after AT, one had bilateral PT after AT as the first disease manifestation. Altogether, our findings suggest for the first time that AT by itself could be a risk factor for PT occurrence in LAM.

The 2010 European Respiratory Society guidelines on LAM provided recommendations regarding AT [41]. LAM patients with minimal respiratory manifestations were not discouraged to fly, unless they presented new respiratory symptoms not evaluated by a physician. Patients with a known untreated PT or a PT treated within the previous month were advised not to travel by air. We believe that our findings should not lead to modify these recommendations, as the additional risk of AT is much smaller than the baseline risk of PT in LAM. However, patients should be informed of this additional risk.

Recently, Johannesma et al. evaluated by questionnaires the risk of spontaneous PT due to AT in patients with BHD, an autosomal genetic disease characterised by skin lesions, renal tumors and multiple pulmonary cysts [11, 15, 18]. From the 145 patients who flew, 13 presented a PT confirmed by chest X-ray ≤1 month after AT. The risk was 0.63% per flight, i.e. lower than in LAM (11, 15, 18, and the present study). Consistently, the frequency of PT during disease course is lower in BHD (35–38%) than in LAM (50–80%) [6–9, 19, 37, 42, 43], a difference possibly explained by less numerous cysts in BHD, or cysts less prone to rupture [18].

The present study has several strengths. We studied a relatively large cohort of patients with LAM from various countries. We considered each lung as an independent observation, thus allowing to analyse particular situations such as bilateral PT. We restricted the analyses to cases with available dates of events and excluded those with missing, incomplete or doubtful dates, even if a PT was narratively reported as occurring after AT (*n* = 3). By using a 30-days interval as the period at risk for PT occurrence after AT, we took into account the fact that PT occurrence and diagnosis may be delayed, as previously reported [17].

Our study has several limitations. As the study design was a patient survey, we did not check the accuracy of the diagnosis. However, since patients were members of LAM associations, we assumed that LAM was the correct diagnosis in all. As study participants were lay persons, one could argue that they did not have enough knowledge to accurately fill the questionnaire. However, participants were not a sample of the general population, but young adults affected by a rare disease, and members of LAM patient associations, which provide regular educational sessions on LAM to their members. These patients were therefore well informed of PT mechanisms, symptoms, diagnosis and therapy. We recorded a maximum of 4 AT and 4 PT episodes, which may have led to underestimate the number of events. However, the number of patients who experienced 4 PT on the same side was small (*n* = 10), and we believe that we did not miss an important proportion of events. Recall bias and errors in questionnaire filling might have occurred. However, we checked by a second questionnaire the validity of the data regarding PT occurring after AT. Patients who replied to the survey may not be fully representative of the whole LAM population. Furthermore, patients who experienced symptoms during AT or feared the occurrence of PT may have been more prone to respond to the survey. The number of events was small, and a larger sample would be required to measure more accurately the risk of PT occurrence after AT. We acknowledge that, even if used in one previous study on PT and AT in BHD [18], the 30-days interval used to define the period at risk of PT after AT was arbitrary. However, shorter intervals would have led to a reduced number of events and an underpowered analysis, whereas longer intervals would have obfuscated AT-related PT among spontaneous PT. Finally, other causes of decreased barometric pressure, such as meteorological changes or ascent to high altitude, were not accounted. Despite these methodological limitations, we believe that our approach provides a valuable new insight in a poorly studied phenomenon.

## Conclusion

The annual incidence of PT in the LAM population was 8% per year since the first symptoms and 5% per year since LAM diagnosis, i.e. around 1000 times higher than the risk of spontaneous PT in the general female population. Pleurodesis after the first PT partially but significantly reduced the risk of subsequent PT. The probability of PT within 30 days after AT was increased threefold as compared to periods without AT, suggesting for the first time that AT by itself could be a risk factor for PT occurrence in LAM. This study also illustrates the valuable role of patient associations in research on rare diseases.

## Abbreviations

AT: air travel
BHD: Birt-Hogg-Dubé syndrome
CI: confidence interval
FEV1: forced expiratory volume in one second
FLAM: France Lymphangioléiomyomatose (French patient association)
LAM: lymphangioleiomyomatosis
PT: pneumothorax
SD: standard deviation
